# A Novel 2-Metagene Signature to Identify High-Risk HNSCC Patients amongst Those Who Are Clinically at Intermediate Risk and Are Treated with PORT

**DOI:** 10.3390/cancers14123031

**Published:** 2022-06-20

**Authors:** Shivaprasad Patil, Annett Linge, Hannah Hiepe, Marianne Grosser, Fabian Lohaus, Volker Gudziol, Max Kemper, Alexander Nowak, Dominik Haim, Inge Tinhofer, Volker Budach, Maja Guberina, Martin Stuschke, Panagiotis Balermpas, Jens von der Grün, Henning Schäfer, Anca-Ligia Grosu, Amir Abdollahi, Jürgen Debus, Ute Ganswindt, Claus Belka, Steffi Pigorsch, Stephanie E. Combs, Simon Boeke, Daniel Zips, Korinna Jöhrens, Gustavo B. Baretton, Michael Baumann, Mechthild Krause, Steffen Löck, on behalf of the DKTK-ROG

**Affiliations:** 1 German Cancer Research Center (DKFZ), 69120 Heidelberg, Germany and German Cancer Consortium (DKTK), partner site Dresden, 01307 Dresden, Germany; shivaprasad309319@gmail.com (S.P.); annett.linge@uniklinikum-dresden.de (A.L.); fabian.lohaus@uniklinikum-dresden.de (F.L.); korinna.joehrens@ukdd.de (K.J.); gustavo.baretton@uniklinikum-dresden.de (G.B.B.); mechthild.krause@uniklinikum-dresden.de (M.K.); 2 German Cancer Research Center (DKFZ), 69120 Heidelberg, Germany, and German Cancer Consortium (DKTK), partner site Berlin, 10117 Berlin, Germany; ingeborg.tinhofer@charite.de (I.T.); volker.budach@charite.de (V.B.); 3 German Cancer Research Center (DKFZ), 69120 Heidelberg, Germany, and German Cancer Consortium (DKTK), partner site Essen, 45147 Essen, Germany; maja.guberina@uk-essen.de (M.G.); martin.stuschke@uk-essen.de (M.S.); 4 German Cancer Research Center (DKFZ), 69120 Heidelberg, Germany, and German Cancer Consortium (DKTK), partner site Frankfurt, 60590 Frankfurt, Germany; panagiotis.balermpas@usz.ch (P.B.); jens.vondergruen@kgu.de (J.v.d.G.); 5 German Cancer Research Center (DKFZ), 69120 Heidelberg, Germany, and German Cancer Consortium (DKTK), partner site Freiburg, 79106 Freiburg, Germany; henning.schaefer@uniklinik-freiburg.de (H.S.); anca.grosu@uniklinik-freiburg.de (A.-L.G.); 6 German Cancer Research Center (DKFZ), 69120 Heidelberg, Germany, and German Cancer Consortium (DKTK), partner site Heidelberg, 69120 Heidelberg, Germany; a.amir@dkfz-heidelberg.de (A.A.); juergen.debus@med.uni-heidelberg.de (J.D.); 7 German Cancer Research Center (DKFZ), 69120 Heidelberg, Germany, and German Cancer Consortium (DKTK), partner site Munich, 80336 Munich, Germany; ute.ganswindt@i-med.ac.at (U.G.); claus.belka@med.uni-muenchen.de (C.B.); steffi.pigorsch@tum.de (S.P.); stephanie.combs@tum.de (S.E.C.); 8 German Cancer Research Center (DKFZ), 60120 Heidelberg, Germany and German Cancer Consortium (DKTK), partner site Tübingen, 72076 Tübingen, Germany; simon.boeke@med.uni-tuebingen.de (S.B.); daniel.zips@med.uni-tuebingen.de (D.Z.); 9 OncoRay—National Center for Radiation Research in Oncology, Faculty of Medicine and UniversityHospital Carl Gustav Carus, Technische Universität Dresden, Helmholtz-Zentrum Dresden—Rossendorf, 01307 Dresden, Germany; hannah.hiepe@uniklinikum-dresden.de (H.H.); michael.baumann@dkfz-heidelberg.de (M.B.); 10 Helmholtz-Zentrum Dresden—Rossendorf, 01307 Dresden, Germany; 11 Department of Radiotherapy and Radiation Oncology, Faculty of Medicine and University Hospital Carl Gustav Carus, Technische Universität Dresden, 01307 Dresden, Germany; 12 National Center for Tumor Diseases (NCT), Partner Site Dresden, Germany: German Cancer Research Center (DKFZ), Heidelberg, Germany; Faculty of Medicine and University Hospital Carl Gustav Carus, Technische Universität Dresden, 01307 Dresden, Germany; Helmholtz Association/Helmholtz-Zentrum Dresden—Rossendorf (HZDR), 01307 Dresden, Germany; max.kemper@uniklinikum-dresden.de (M.K.); alexander.nowak@uniklinikum-dresden.de (A.N.); dominik.haim@uniklinikum-dresden.de (D.H.); 13 Institute of Pathology, Faculty of Medicine and University Hospital Carl Gustav Carus, Technische Universität Dresden, 01307 Dresden, Germany; marianne.grosser@uniklinikum-dresden.de; 14 Department of Otorhinolaryngology, Faculty of Medicine and University Hospital Carl Gustav Carus, Technische Universität Dresden, 01307 Dresden, Germany; volker.gudziol@uniklinikum-dresden.de; 15 Department of Otorhinolaryngology, Head and Neck Surgery, Municipal Hospital Dresden, 01067 Dresden, Germany; 16 Department of Oral and Maxillofacial Surgery, Faculty of Medicine and University Hospital Carl Gustav Carus, Technische Universität Dresden, 01307 Dresden, Germany; 17 Department of Radiooncology and Radiotherapy, Charité University Medicine Berlin, 10117 Berlin, Germany; 18 Department of Radiation Therapy, University Hospital, Medical Faculty, University of Duisburg-Essen, 45147 Essen, Germany; 19 Department of Radiotherapy and Oncology, Goethe-University Frankfurt, 60590 Frankfurt, Germany; 20 Department of Radiation Oncology, Medical Center, Medical Faculty, University of Freiburg, 79106 Freiburg, Germany; 21 Heidelberg Institute of Radiation Oncology (HIRO), National Center for Radiation Research in Oncology (NCRO), University of Heidelberg Medical School and German Cancer Research Center (DKFZ), 69120 Heidelberg, Germany; 22 Heidelberg Ion Therapy Center (HIT), Department of Radiation Oncology, University of Heidelberg Medical School, 69120 Heidelberg, Germany; 23 National Center for Tumor Diseases (NCT), University of Heidelberg Medical School and German Cancer Research Center (DKFZ), 69120 Heidelberg, Germany; 24 Translational Radiation Oncology, University of Heidelberg Medical School and German Cancer Research Center (DKFZ), 69120 Heidelberg, Germany; 25 Clinical Cooperation Unit Radiation Oncology, University of Heidelberg Medical School and German Cancer Research Center (DKFZ), 69120 Heidelberg, Germany; 26 Department of Radiotherapy and Radiation Oncology, University Hospital, Ludwig-Maximilians-Universität, 81377 Munich, Germany; 27 Clinical Cooperation Group Personalized Radiotherapy in Head and Neck Cancer, Helmholtz Zentrum Munich, 85764 Neuherberg, Germany; 28 Department of Radiation Oncology, Technische Universität München, 81675 Munich, Germany; 29 Department of Radiation Sciences (DRS), Institut für Innovative Radiotherapie (iRT), Helmholtz Zentrum Munich, 85764 Neuherberg, Germany; 30 Department of Radiation Oncology, Faculty of Medicine and University Hospital Tübingen, Eberhard Karls Universität Tübingen, 72076 Tübingen, Germany; 31 Tumor- and Normal Tissue Bank, University Cancer Centre (UCC), University Hospital Carl Gustav Carus, Technische Universität Dresden, 01307 Dresden, Germany; 32 German Cancer Research Center (DKFZ), 69120 Heidelberg, Germany

**Keywords:** head and neck squamous cell carcinoma, gene signature, postoperative radiotherapy, postoperative radiochemotherapy, propensity score matching

## Abstract

**Simple Summary:**

The aim of this matched-pair study including patients with locally advanced head and neck squamous cell carcinoma (HNSCC) was to identify patients who are biologically at high risk for the development of loco–regional recurrences after surgery and postoperative radiotherapy (PORT) but at intermediate risk according to clinical risk factors, with the help of a novel predictive gene signature. These patients may benefit from treatment with postoperative radiochemotherapy (PORT-C). Based on 108 matched patient pairs treated with PORT and PORT-C, we identified a gene signature consisting of two metagenes. A significant association of the interaction between the risk classification by this signature and the type of treatment was observed for the endpoint loco–regional control (LRC), i.e., the 2-metagene signature was indicative for the type of treatment. The developed signature may thus help to identify high-risk patients currently treated with PORT, who may benefit from additional concurrent chemotherapy.

**Abstract:**

(1) Background: Patients with locally advanced head and neck squamous cell carcinoma (HNSCC) who are biologically at high risk for the development of loco–regional recurrences after postoperative radiotherapy (PORT) but at intermediate risk according to clinical risk factors may benefit from additional concurrent chemotherapy. In this matched-pair study, we aimed to identify a corresponding predictive gene signature. (2) Methods: Gene expression analysis was performed on a multicenter retrospective cohort of 221 patients that were treated with postoperative radiochemotherapy (PORT-C) and 283 patients who were treated with PORT alone. Propensity score analysis was used to identify matched patient pairs from both cohorts. From differential gene expression analysis and Cox regression, a predictive gene signature was identified. (3) Results: 108 matched patient pairs were selected. We identified a 2-metagene signature that stratified patients into risk groups in both cohorts. The comparison of the high-risk patients between the two types of treatment showed higher loco–regional control (LRC) after treatment with PORT-C (*p* < 0.001), which was confirmed by a significant interaction term in Cox regression (*p* = 0.027), i.e., the 2-metagene signature was indicative for the type of treatment. (4) Conclusion: We have identified a novel gene signature that may be helpful to identify patients with high-risk HNSCC amongst those at intermediate clinical risk treated with PORT, who may benefit from additional concurrent chemotherapy.

## 1. Introduction

Patients with locally advanced head and neck squamous cell carcinoma (HNSCC) are currently treated with primary radiotherapy, surgery followed by postoperative radiotherapy (PORT), or postoperative radiochemotherapy (PORT-C), depending on the clinical characteristics of the tumour [1,2]. Several studies and randomized controlled trials have demonstrated the benefits of concurrent radiochemotherapy over radiotherapy alone in patients with locally advanced HNSCC, showing improved local recurrence and disease-free survival with a manageable increase in toxicity [3,4,5,6,7,8,9]. Brizel et al. showed that patients with high-risk HNSCC demonstrated a significant improvement in loco–regional control and disease-free survival when treated with concurrent postoperative radiochemotherapy [7]. Another study by Bernier et al. found that concurrent radiochemotherapy improved progression-free survival from 36% to 47% and overall survival from 40% to 53% in comparison to radiotherapy alone in patients with locally advanced HNSCC [10].

Postoperative radiotherapy is commonly applied in patients with intermediate risk factors, often characterized by large tumours and none or few positive lymph nodes without or very little (<1 mm) extracapsular extension [11,12,13,14,15,16]. Additional concurrent chemotherapy is usually indicated in case of further clinical risk factors such as ≥2 positive lymph nodes, extracapsular extension of lymph node metastases, microscopic disease after surgery (R1 resection), and UICC stages III-IV [7,10,17]. Still, treatment outcome after PORT is heterogeneous with a local recurrence rate after two years of around 38% [18], i.e., some patients may be judged to be clinically at intermediate risk but actually are at high risk for the development of a recurrence and may benefit from the addition of concurrent chemotherapy.

In the past decade, gene expression data have been used to identify prognostic and predictive gene signatures that predict recurrence and response to therapy [19,20,21,22,23,24,25]. For example, the 15-gene hypoxia-associated signature [20], which consists of upregulated genes under hypoxic conditions [26], proved to be a useful predictive biomarker for the selection of patients with HNSCC that benefit from hypoxic modification of primary radiotherapy with nimorazole. Similarly, a 22-gene signature based on TCGA data showed that patients with HNSCC classified as high-risk who received radiochemotherapy demonstrated improved overall survival, relapse-free survival, and loco–regional control compared with those patients that received radiotherapy alone [27]. However, this study did not account for differences in clinical characteristics between patients who received PORT and PORT-C, which may induce a selection bias.

Therefore, in the present study, we aimed to develop a novel predictive gene signature to identify a subgroup of patients treated with PORT who were clinically judged to be at intermediate risk but actually had a high risk for loco–regional failure and may benefit from additional concurrent chemotherapy. Based on whole-transcriptome data, we performed a propensity score matched analysis of two retrospective datasets of patients with locally advanced HNSCC treated with PORT and PORT-C.

## 2. Materials and Methods

### 2.1. Patient Data

**Figure 1:**
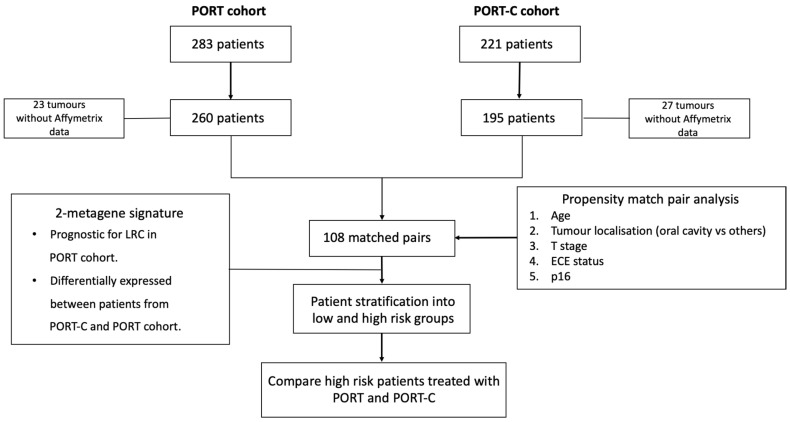
Study design.

In this retrospective study, two cohorts with locally advanced HNSCC were included. In total, 221 patients were treated with PORT-C between 2004 and 2012 in 9 different institutions of the German Cancer Consortium—Radiation Oncology Group (DKTK-ROG) [28] and 283 patients were treated with PORT between 1999 and 2016 at the DKTK-ROG site in Dresden. All patients received surgery followed by postoperative radio(chemo)therapy and met the following inclusion criteria: histologically proven squamous cell carcinoma, curatively intended cisplatin-based PORT-C or PORT according to standard protocols covering the former tumour region and the neck nodes. Patients were excluded if whole-transcriptome data were not available, reducing the patient number to 195 in the PORT-C cohort and to 260 in the PORT cohort. Additional details on inclusion criteria, data collection, handling, and analyses of biomaterial have been described previously [28,29]. In this study, the 7th edition (2010) of the TNM classification has been used. The study design is illustrated in Figure 1.

The treating institution evaluated the disease status and first site of relapse. The radiotherapy treatment plan and radiological images of the recurrence (CT, MRI or PET-CT) for each loco–regional failure were reviewed by experienced radiation oncologists. FFPE blocks of the resected tumour specimens were collected centrally at the DKTK partner site in Dresden. Total RNA extraction was performed as described previously [28,29]. The CINtec Histology kit (Roche mtm laboratories AG, Basel, CH) was used to perform immunohistochemical staining of p16, according to the manufacturer’s instructions as described previously [28,29]. Tumours with intense p16 nuclear staining in at least 70% of the tumour cells were considered as p16 overexpressing. The ethical approval for the multicenter retrospective analyses of clinical and biological data were obtained by all the DKTK-ROG partner sites.

### 2.2. Microarray Analysis

The Human Transcriptome 2.0 Array (Thermo Fisher Scientific Inc., Waltham, MA, USA) was used to perform the whole transcriptome analysis as described previously [30]. Quality control was performed in Transcriptome Analysis Console (TAC) 4.0 (Applied Biosystems, Waltham, MA, USA) as per manufacturer’s instructions using the probe-level intensity files. The Signal Space Transformation in conjunction with the Robust Multiarray Average method (SST-RMA) were used to perform data normalization. Batch normalization was performed using ComBat method [31] to adjust for batch effects between the cohorts since the data were collected during different time intervals. For further analysis, coding genes were selected.

### 2.3. Clinical Endpoints and General Statistical Analysis

The primary endpoint was loco–regional control (LRC), which was calculated from the first day of radiotherapy to the date of event or censoring. Overall survival (OS) and freedom from distant metastases (DM) were the secondary endpoints. Survival curves were estimated using the Kaplan–Meier method and were compared using log-rank tests. To examine differences in continuous and categorical variables between the cohorts, Mann–Whitney-U tests and chi-squared (χ^2^) tests were used, respectively. To test the association of the genes and clinical features with the endpoints, univariable Cox regression was used. R Statistics version 3.6.1 (R Foundation for Statistical Computing, Vienna, Austria) [32], Python (Python Software Foundation. Python Language Reference, version 3.7) and SPSS 25 software (IBM Corporation, Armonk, NY, USA) were used to perform the described statistical tests. Two-sided tests were performed and *p*-values < 0.05 were considered as statistically significant for all analyses.

### 2.4. Matched-Pair Analysis

The exposure of interest was whether patients received PORT or PORT-C (treatment status). Logistic regression was used to estimate a propensity score model, where the treatment status was regressed on the clinical variables of age (0: <57 years vs. 1: ≥57 years, based on median), T stage (1, 2 vs. 3, 4), tumour localization (oral cavity vs. others), extracapsular extension (ECE) status (0 vs. 1), and p16 overexpression (0 vs. 1). These parameters were significantly associated with LRC in at least one of the cohorts (Table S1). Pairs of patients treated with PORT and PORT-C were matched using the nearest method on the logit propensity score using different caliper widths. An optimal caliper width of 0.2 was chosen based on the standard mean square difference between the clinical parameters in PORT and PORT-C patients after propensity score matching and based on the number of matched patient pairs. The analysis was performed using the matchIt R package [33].

### 2.5. Statistical Framework to Identify Gene Signature and Perform Model Predictions

Before identifying the gene signature, the gene expression data of the PORT cohort were z-transformed to mean 0 and standard deviation of 1. Based on the obtained mean values and standard deviations, the corresponding gene expression data of the PORT-C cohort were transformed.

To identify a predictive gene signature, the following steps were carried out. (i) Univariable Cox regression analysis was performed on the PORT cohort to filter genes with high prognostic value for LRC. (ii) Differential gene expression (DGE) analysis was performed between the PORT-C cohort and the PORT cohort to identify genes that represent a differing response between both cohorts. The genes with a fold-change (FC) of ≥1.5 and with FDR corrected *p*-values of ≤0.05 in differential gene expression and univariable Cox regression were selected. To increase robustness of the signature, genes that were highly correlated in the PORT cohort (Spearman correlation coefficient r ≥ 0.8) were combined to create a new metagene, defined as the median expression of the contributing genes. Finally, the identified genes or metagenes were used to build a multivariable Cox model. The risk score of each patient was calculated as: ∑ coefficient of the feature in multivariable Cox model (β_i_) × value of the feature. An optimal risk score for patient stratification was calculated using the maximally selected rank statistics (maxstat) R package [34] based on 1000 bootstraps. The optimal risk score from the PORT cohort was used as a cut-off for stratifying patients into high-risk and low-risk groups in both the PORT and PORT-C cohort and thereby defines a corresponding gene classifier. Finally, the high-risk groups of both cohorts were compared by a log-rank test and an interaction term between treatment type and the expression of the gene signature was considered in Cox regression to test the predictive value of the signature. An experienced biostatistician (S.L) guided the statistical analyses.

## 3. Results

Patient data and clinical parameters of both cohorts before matching are summarized in Table S2. In the PORT cohort, 52.3% of the patients presented with oral cavity carcinomas, while this was the case for only 28.2% of patients in the PORT-C cohort (*p* < 0.001). A higher number of patients in the PORT-C cohort were associated to high T-stage (*p* = 0.038) and N-stage (*p* < 0.001). Patients in the PORT cohort had lower LRC than patients in the PORT-C cohort (*p* = 0.082), while OS (*p* = 0.24) and DM (*p* = 0.16) were similar (Figure S1).

After propensity score matching, 108 matched patient pairs were obtained. Standardized mean differences for the five clinical parameters were smaller than 0.1 (Table S3). Patient data and clinical parameters of the matched cohorts are summarized in Table 1. After matching, patients in the PORT cohort had lower LRC (*p* = 0.037) than patients in the PORT-C cohort, while OS (*p* = 0.12) and DM (*p* = 0.42) were similar (Figure S2). 

Seven genes, *KRT6A*, *KRT6B*, *KRT6C*, *SPRR1A*, *SPRR1B*, *SPRR2A*, and *SPRR2C*, that were differentially expressed between the matched cohorts and prognostic on the PORT cohort, were identified. Due to their high correlation (r > 0.80), two metagenes were formed based on *KRT6A*, *KRT6B*, and *KRT6C* and based on *SPRR1A*, *SPRR1B*, *SPRR2A*, and *SPRR2C*; finally defining the proposed predictive 2-metagene signature.

**Figure 2:**
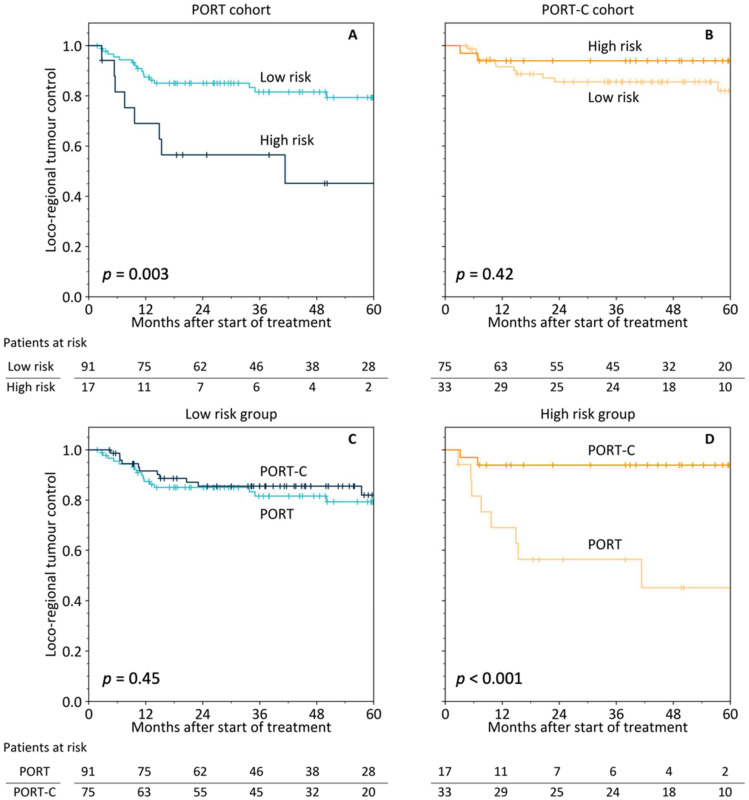
Patient stratification by the 2-metagene signature for loco–regional tumour control (LRC) in the PORT (**A**) and the PORT-C cohort (**B**). Comparison of LRC between PORT and PORT-C for the low-risk (**C**) and high-risk (**D**) groups as defined by the 2-metagene signature.

From the multivariable Cox regression model on the PORT cohort, an individual risk score (rs) was calculated for every patient: rs=0.374× metageneKRT6+0.266× metageneSPRR. Upregulation of both metagenes was related to reduced LRC. Patient stratification using the optimal risk score cut-off of 0.60 led to a significant difference in LRC for the PORT cohort (*p* = 0.003) but not for the PORT-C cohort (*p* = 0.42), Figure 2A,B. Comparing the patients classified to be at high risk between the two treatment types revealed a significant benefit of PORT-C (*p* < 0.001), while there was no difference between the patients classified to be at low risk (*p* = 0.45), Figure 2C,D. A multivariable model including the 2-metagene-classifier, treatment status, and their interaction term (gene classifier × treatment status) showed a statistically significant interaction term (*p* = 0.027, Table 2), i.e., the signature was predictive for the type of treatment. The interaction term remained significant when additional clinical characteristics were included in the model (*p* = 0.023, Table 2).

Concerning the secondary endpoints, the 2-metagene signature was prognostic for OS in the PORT cohort but not for DM. Kaplan–Meier curves of patient groups stratified by the 2-metagene signature for OS and DM are presented in Figure S3 for both cohorts. The multivariable models including the 2-metagene-classifier, treatment status, and their interaction term (gene classifier × treatment status) did not show a significant interaction term. The results are presented in Tables S4 and S5, respectively.

## 4. Discussion

In this matched-pair study, we developed a novel 2-metagene signature based on differential gene expression analysis and Cox regression in order to identify HNSCC patients amongst those who are biologically at high risk for the development of loco–regional recurrences after postoperative radiotherapy (PORT) but clinically considered to be at intermediate risk. We showed that patients classified as high risk who were treated with PORT-C had significantly higher LRC compared to similar patients treated with PORT.

The genes *KRT6A*, *KRT6B*, and *KRT6C* encode a type II cytokeratin that is important in the formation of nail bed, filiform papillae, and the epithelial lining of oral mucosa and the esophagus. *KRT6A* gene silencing has been shown to suppress cell viability, invasion, and metastasis of nasopharyngeal carcinoma via the β-catenin/TCF pathway [35]. *KRT6A* is also overexpressed in lung adenocarcinoma and promotes lung cancer cell proliferation, migration, and colony formation ability via epithelial–mesenchymal transition and cancer stem cells transformation [36]. The overexpression of *KRT6B* has been shown to significantly suppress honokiol-induced human hepatoma cell apoptosis via notch signaling [37]. A 25-gene network signature model by Chang et al. was able to discriminate between two histological types of lung cancers, adenocarcinomas and squamous cell carcinomas, and 95% of the accuracy was explained by the interplay of *KRT6A*, *KRT6B*, and *KRT6C*, which were unique to squamous cells [38].

The *SPRR* genes encode a class of polypeptides (small proline rich proteins) that are involved in differentiation of keratinocytes, the primary cell type of the epidermis. *SPRR1A* is known to play a role in various types of cancer, such as diffuse large B-cell lymphomas [39], head and neck squamous cell carcinoma [40], and breast cancer [41]. The overexpression of *SPRR1B* has been shown to enhance the entry of cells in the G0 phase of the cell cycle [42]. *SPRR1B* is also known to be overexpressed in human oral squamous cell cancer stem-like cells and is related to their growth through activation of MAP kinase signal [43]. *SPRR2A* is overexpressed in lymph node metastases, along with an association to non-oropharyngeal location of the primary tumour and is an independent prognostic factor for regional disease recurrence after surgery and radiotherapy. It plays a dual role in invasion and therapeutic resistance in HNSCC, respectively through its downregulation and overexpression [44].

Studies on gene ontology revealed that the seven identified genes were enriched in biological processes such as keratinocyte differentiation, epithelial differentiation, and skin development. All genes and both metagenes showed positive coefficients in the univariable Cox model suggesting that overexpression was associated with worse prognosis in HNSCC, which is in line with the literature [35,36,37,38,39,40,41,42,43,44].

The clinical characteristics between the two considered patient cohorts are expected to differ, since patients with different clinical risk profiles were included. Significant differences were observed in age, radiation dose, tumour localization, R status, ECE status, T stage, and N stage between the PORT and PORT-C cohorts before matching (Table S2). After matching with five clinical characteristics, most clinical parameters were well aligned between the cohorts, except for dose, R status, and N stage. However, none of these parameters were significantly related to LRC (Table 3). Propensity score matching with additional clinical parameters led to too small patient numbers in the matched cohorts and was thus not considered.

We showed that the 2-metagene signature may be used as a predictive biomarker to select HNSCC patients who are clinically considered at intermediate risk but may benefit from additional chemotherapy as a treatment intensification strategy. Further intensification of PORT-C was investigated in a phase II trial, where it was suggested that adding panitumumab, an antibody to *EGFR*, might be superior to PORT-C for high risk HPV-negative HNSCC patients [45]. In our PORT cohort, *EGFR* was found to be prognostic for LRC (*p* = 0.007), a high expression was related to worse outcome. In general, other prognostic biomarkers may also be considered for treatment intensification. Several genes or gene signatures have previously been identified [19,20,21,22,23,24,25]. On the PORT cohort, the potential stem cell marker *SLC3A2* [19], the 6-gene signature associated to cell migration and invasion [46], the 12-gene immune signature [23], and the 15-gene hypoxia-associated signature [20] were prognostic for LRC. Therefore, the molecular pathways associated with these genes or gene signatures may contain potential targets for treatment intensification purposes.

There are several limitations to the study. First, this study is retrospective in nature, and although propensity score matching was performed, bias inevitably exists due to the exclusion of patients as no match could be found using the nearest method. After matching, from 195 patients treated with PORT-C and 260 treated with PORT, we were able to include 108 matched patient pairs, where the standard mean difference among the matched clinical features was smaller than 0.1 (Table S3). The obtained results need to be externally and prospectively validated. This is planned by using data from the prospective HNprädBio trial (NCT02059668, www.clinicaltrials.gov (accessed on 2 February 2022)) of the DKTK-ROG that will finish patient recruitment in 2022.

## 5. Conclusions

In conclusion, we identified a novel 2-metagene signature that may be used to identify high-risk HNSCC patients amongst those who are clinically at intermediate risk and, according to current guidelines, treated with PORT. These patients may benefit from treatment with additional concurrent chemotherapy. Independent prospective validation of this retrospective result is required before potential application in a clinical trial.

## Figures and Tables

**Table 1:** Patient characteristics for the PORT and PORT-C cohorts (108 matched patient pairs). Significant *p*-values are marked in bold.

Characteristics	PORT Cohort (1999–2016)		PORT-C Cohort (2004–2011)		*p*-Value
	Median (Range)		Median (Range)		
Age (years)	57.3 (39.0–84.3)		57 (24–74)		0.26
Dose (Gy)	60.0 (60–66)		64.0 (56–68.4)		**<0.001**
	**Number of pts**	**%**	**Number of pts**	**%**	
Age 0(<57)/1(≥57 years)	51/57	47.2/52.8	53/55	49.1/50.9	0.79
Gender Male/female	90/18	88.3/11.7	88/20	81.5/18.5	0.72
Tumour localization Oral cavity/Oropharynx/Hypopharynx/Larynx	31/55/6/16	28.7/50.9/5.5/14.9	31/59/18/0	28.7/54.6/16.7/0	1.00
Grading 1,2/3	51/57	47.2/52.8	45/63	41.7/58.3	0.10
R status 0/1/missing	100/8	92.6/7.4	59/49	54.6/45.4	**<0.001**
ECE status 0/1/missing	85/23	78.7/21.3	85/23	78.7/21.3	1.00
p16 overexpression 0/1	71/37	65.7/34.3	67/41	62.0/38.0	0.57
T stage 1,2/3,4	74/34	68.5/31.5	69/39	63.9/36.1	0.47
N stage 0,1/2,3	68/40	63.0/37.0	37/71	38.0/62.0	**<0.001**
Locoregional control	25	23.1	14	13.0	**0.037** ^a^
Distant metastases	19	17.6	15	13.9	0.42 ^a^
Overall survival	46	42.6	31	28.7	0.12 ^a^

^a^ Log-rank test.

**Table 2:** Multivariable Cox regression of loco–regional tumour control for the 2-metagene signature, treatment, their interaction term, and relevant clinical parameters for the pooled matched dataset (*n* = 216). Significant *p*-values are marked in bold.

Parameter	Coefficient (ß)	Loco–Regional Control HR (95 % CI)	*p*-Value
**2-Gene signature**			
Gene classifier (high vs. low risk [b])	1.22	3.42 (1.47–7.97)	**0.004**
Treatment status (PORT-C vs. PORT [b])	−0.30	0.74 (0.35–1.58)	0.44
Gene classifier × Treatment status	−1.73	0.18 (0.04–0.82)	**0.027**
**2-Gene signature and clinical parameters**			
Gene classifier (high vs. low risk [b])	1.19	3.29 (1.37–7.91)	**0.007**
Treatment status (PORT-C vs. PORT [b])	−0.57	0.56 (0.24–1.33)	0.19
Gene classifier × Treatment status	−1.81	0.16 (0.03–0.78)	**0.023**
T stage (3, 4 vs. 1, 2 [b])	0.99	2.68 (1.33–5.40)	**0.005**
Tumour localization (oral cavity vs. others [b])	0.58	1.79 (0.88–3.64)	0.11
N stage (2, 3 vs. 0, 1 [b])	0.38	1.46 (0.65–3.27)	0.36
R status (1 vs. 0 [b])	0.40	1.49 (0.69–3.30)	0.33
ECE status (1 vs. 0 [b])	0.48	1.61 (0.66–3.92)	0.29
p16 overexpression (1 vs. 0 [b])	−0.97	0.38 (0.15–0.94)	**0.037**

[b] Baseline class.

**Table 3:** Univariable Cox regression of loco–regional tumour control for clinical parameters and the identified two metagenes in the matched PORT and PORT-C cohorts (*n* = 108). Significant *p*-values are marked in bold.

Parameter	PORT Cohort			PORT-C Cohort		
	Coefficient (ß)	Loco–Regional Control HR (95 % CI)	*p*-Value	Coefficient (ß)	Loco–Regional Control HR (95 % CI)	*p*-Value
Age (≥57 vs. <57 years [b])	−0.75	0.47 (0.21–1.07)	0.074	−1.54	0.21 (0.06–0.78)	**0.019**
Gender (female vs. male [b])	0.11	1.12 (0.41–3.04)	0.83	0.67	1.96 (0.61–6.26)	0.26
Tumour localization (oral cavity vs. others [b])	1.09	2.97 (1.34–6.60)	**0.007**	0.55	1.73 (0.58–5.21)	0.33
T stage (3, 4 vs. 1, 2 [b])	1.13	3.10 (1.41–6.81)	**0.004**	0.55	1.73 (0.60–5.03)	0.31
N stage (2, 3 vs. 0, 1 [b])	0.42	1.55 (0.69–3.38)	0.30	−0.01	0.99 (0.33–2.97)	0.98
Tumour grade (3 vs. 1, 2 [b])	−0.33	0.72 (0.33–1.58)	0.41	−0.96	0.38 (0.11–1.37)	0.14
R status (1 vs. 0 [b])	0.95	2.58 (0.88–7.60)	0.085	0.09	1.10 (0.38–31.8)	0.87
ECE status (1 vs. 0 [b])	0.83	2.29 (0.98–5.32)	0.055	0.24	1.27 (0.35–4.62)	0.72
Dose (Gy)	0.07	1.08 (0.93–1.25)	0.32	0.07	1.08 (0.87–1.33)	0.51
p16 overexpression (1 vs. 0 [b])	−1.20	0.30 (0.10–0.88)	**0.029**	−1.38	0.25 (0.06–1.13)	0.071
Metagene *KRT6*	0.59	1.80 (1.17–2.79)	**0.008**	0.49	1.62 (0.87–3.02)	0.13
Metagene *SPRR1*	0.49	0.57 (0.34–0.98)	**0.004**	0.21	1.23 (0.82–1.86)	0.32

[b] Baseline class.

## Data Availability

The data presented in the study are available on request from the corresponding author. The data are not publicly available due to further ongoing analyses.

## Supplementary Materials

The following supporting information can be downloaded at: https://www.mdpi.com/article/10.3390/cancers14123031/s1, Figure S1: Loco–regional tumour control (A), overall survival (B) and freedom from distant metastases (C) on the PORT and PORT-C cohort before propensity score matching; Figure S2: Loco–regional tumour control (A), overall survival (B) and freedom from distant metastases (C) on the PORT and PORT-C cohort after propensity score matching; Figure S3: Patient stratification by the 2-metagene signature for overall survival (OS) in the PORT (A) and the PORT-C cohort (B) and for freedom from distant metastases (DM) in the PORT (C) and the PORT-C cohort (D); Table S1: Univariable Cox regression of loco–regional tumour control for the clinical parameters in the PORT and PORT-C cohorts before propensity score matching.; Table S2: Patient characteristics for the PORT and PORT-C cohorts before propensity score matching; Table S3: Comparison of the clinical parameters between PORT and PORT-C patients in the original sample and in the propensity score matched sample. The standardized mean and the standardized mean difference between the cohorts are shown. Table S4: Multivariable Cox regression of overall survival for the 2-metagene signature and their interaction term with treatment type; Table S5: Multivariable Cox regression of freedom from distant metastases for the 2-metagene signature and their interaction term with treatment type.
