# Supplementary material for: A Novel 2-Metagene Signature to Identify High-Risk HNSCC Patients amongst Those Who Are Clinically at Intermediate Risk and Are Treated with PORT
Source: Cancers (Basel). 2022 Jun 20;14(12):3031. doi: 10.3390/cancers14123031 (PMC9221048; doi:10.3390/cancers14123031)
Supplement: Supplementary file 1 [file cancers-14-03031-s001.zip › cancers-1732589-supplementary.pdf]

Supplementary Material

A Novel 2-Metagene Signature to Identify High-Risk HNSCC Patients amongst Those Who Are Clinically at Intermediate Risk and Are Treated with PORT

Table S1. Univariable Cox regression of loco-regional tumour control for the clinical parameters in the PORT and PORT-C cohorts before propensity score matching.

	PORT cohort			PORT-C cohort		
**Parameter**	**Coefficient (ß)**	**Loco-regional control HR (95 % CI)**	**p-value**	**Coefficient (ß)**	**Loco-regional control HR (95 % CI)**	**p-value**
**Clinical parameters**						
Age (≥57 vs <57 years [b])	-0.61	0.54 (0.32-0.94)	**0.028**	-0.97	0.38 (0.17-0.83)	**0.016**
Gender (female vs male[b])	0.04	1.04 (0.56-1.94)	0.90	0.49	1.63 (0.72-3.69)	0.28
Tumour localization (oral cavity vs others[b])	0.71	2.03 (1.17-3.53)	**0.015**	1.28	3.62 (1.73-7.57)	**<0.001**
T stage (3,4 vs 1,2[b])	0.88	2.41 (1.41-4.13)	**0.001**	0.96	2.62 (1.25-5.46)	**0.011**
N stage (2,3 vs 0,1[b])	0.43	1.54 (0.90-2.62)	0.11	0.26	1.30 (0.53-3.19)	0.57
Tumour grade (3 vs 1,2[b])	-0.32	0.73 (0.42-1.25)	0.25	-0.61	0.55 (0.24-1.23)	0.15
R status (1 vs 0[b])	0.17	1.18 (0.50-2.78)	0.70	0.04	1.05 (0.50-2.18)	0.91
ECE status (1 vs 0[b])	0.86	2.37 (1.15-5.03)	**0.025**	0.43	1.54 (0.73-3.27)	0.26
Dose (Gy)	0.07	1.08 (0.97-1.19)	0.17	0.10	1.11 (0.93-1.32)	0.23
p16 overexpression (1 vs 0[b])	-1.31	0.27 (0.01-0.75)	**0.012**	-1.49	0.27 (0.08-0.65)	**0.006**

[b] Baseline class.

Table S2. Patient characteristics for the PORT and PORT-C cohorts before propensity score matching.

Characteristics	PORT cohort (1999-2016)		PORT-C cohort (2004-2011)		p-value
	**Median(range)**		**Median(range)**		
Age (years)	57.7 (27.7-88.8)		57 (24-75)		**<0.001**
Dose (Gy)	60.0 (60-66)		64.0 (56-68.4)		**<0.001**
	**S**	**%**	**Number of pts**	**%**	
Gender<br>Male/female	99/59	77.1/22.9	156/39	80.0/20.0	0.46
Tumour localization<br>Oral cavity/<br>Oropharynx/Hypopharynx/Larynx	135/88/13/22	52.3/34.1/5.1/8.5	55/115/25/0	28.2/59.0/12.8/0	**<0.001**
Grading<br>1,2/3	133/124/1	51.6/48.1/0.3	78/117	40.0/60.0	0.098
R status<br>0/1/missing	230/21/7	89.2/8.1/2.7	85/109/1	43.6/55.9/0.5	**<0.001**
ECE status<br>0/1/missing	233/24/1	90.3/9.3/0.4	89/106/0	45.6/44.4	**<0.001**
p16 overexpression<br>0/1/missing	204/51/3	79.1/19.8/1.1	118/73/4	60.5/37.4/2.1	**<0.001**
T stage<br>1,2/3,4	186/72	72.1/27.9	72/123	36.9/63.1	**0.038**
N stage<br>0,1/2,3	167/91	64.7/35.3	47/148	24.1/75.9	**<0.001**
Locoregional control	55	21.3	29	14.9	0.082[a]
Distant metastases	113	43.8	63	32.3	0.24[a]
Overall survival	34	13.2	37	18.9	0.16[a]

[a]Log-rank test

Table S3. Comparison of the clinical parameters between PORT and PORT-C patients in the original sample and in the propensity score matched sample. The standardized mean and the standardized mean difference between the cohorts are shown.

	Original sample			Matched sample (nearest method)		
**Clinical parameter**	**Mean PORT-C (193)**	**Mean PORT (257)**	**Mean difference**	**Mean PORT-C (108)**	**Mean PORT (108)**	**Mean difference**
Distance*	0.617	0.288	1.212	0.440	0.433	0.0272
Age (≥57 vs <57 years)	0.513	0.524	-0.021	0.509	0.528	-0.037
Tumour localization (oral cavity/others)	0.288	0.528	-0.529	0.287	0.287	0.000
T stage (3,4 vs 1,2)	0.377	0.276	0.209	0.361	0.315	0.096
ECE status (1 vs 0)	0.545	0.091	0.912	0.213	0.213	0.000
p16 overexpression (1 vs 0)	0.382	0.201	0.373	0.380	0.343	0.076

*Distance between propensity scores of PORT and PORT-C patients.

Table S4. Multivariable Cox regression of overall survival for the 2-metagene signature and their interaction term with treatment type.

Parameter	Coefficient (ß)	HR (95 % CI)	p-value
**Overall survival**			
Gene classifier (high vs low risk [b])	0.60	1.81 (1.00-3.29)	**0.049**
Treatment status (PORT-C vs PORT [b])	-0.16	0.85 (0.43-1.68)	0.64
Gene classifier * Treatment	-0.53	0.59 (0.23-1.49)	0.26

[b] Baseline class.

Table S5. Multivariable Cox regression of freedom from distant metastases for the 2-metagene signature and their interaction term with treatment type.

Parameter	Coefficient (ß)	HR (95 % CI)	p-value
**Freedom from distant metastases**			
Gene classifier (high vs low risk [b])	0.61	1.85 (0.75-4.57)	0.18
Treatment status (PORT-C vs PORT [b])	0.005	1.01 (0.41-2.48)	0.99
Gene classifier * Treatment	-0.65	0.52 (0.13-2.14)	0.37

[b] Baseline class.

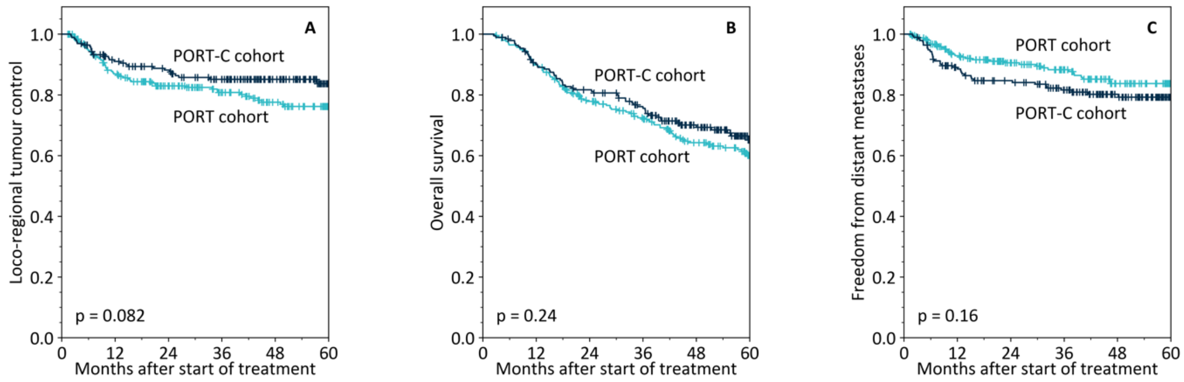

Patients at risk

	0	12	24	36	48	60
PORT cohort	258	208	172	139	111	75
PORT-C cohort	195	164	148	125	88	47

Patients at risk

	0	12	24	36	48	60
PORT cohort	258	230	185	155	124	88
PORT-C cohort	195	171	153	131	91	48

Patients at risk

	0	12	24	36	48	60
PORT cohort	258	212	175	145	113	76
PORT-C cohort	195	161	145	123	87	45

Figure S1. Loco-regional tumour control **(A)**, overall survival **(B),** and freedom from distant metastases **(C)** on the PORT and the PORT-C cohort before propensity score matching.

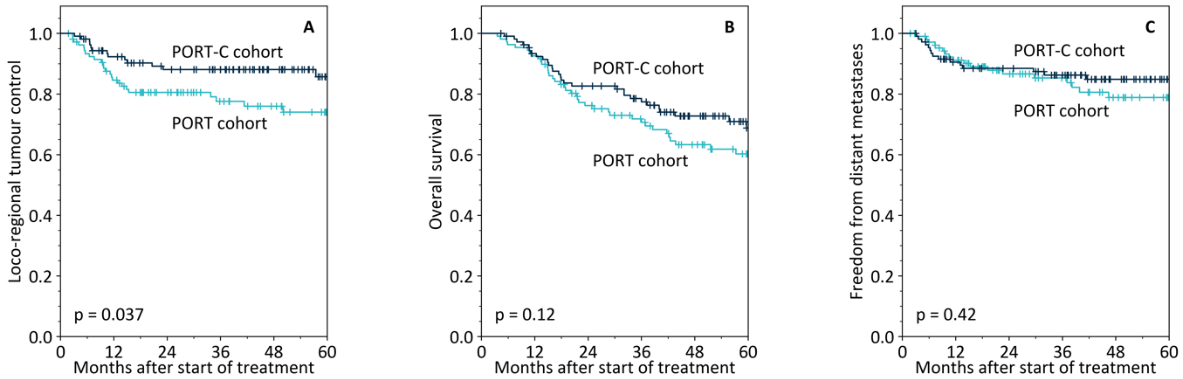

Patients at risk

	0	12	24	36	48	60
PORT cohort	108	86	69	52	42	30
PORT-C cohort	108	92	80	69	50	30

Patients at risk

	0	12	24	36	48	60
PORT cohort	108	100	75	60	47	35
PORT-C cohort	108	96	84	71	50	31

Patients at risk

	0	12	24	36	48	60
PORT cohort	108	90	71	57	43	31
PORT-C cohort	108	89	82	70	49	29

Figure S2. Loco-regional tumour control **(A)**, overall survival **(B),** and freedom from distant metastases **(C)** on the PORT and the PORT-C cohort after propensity score matching.

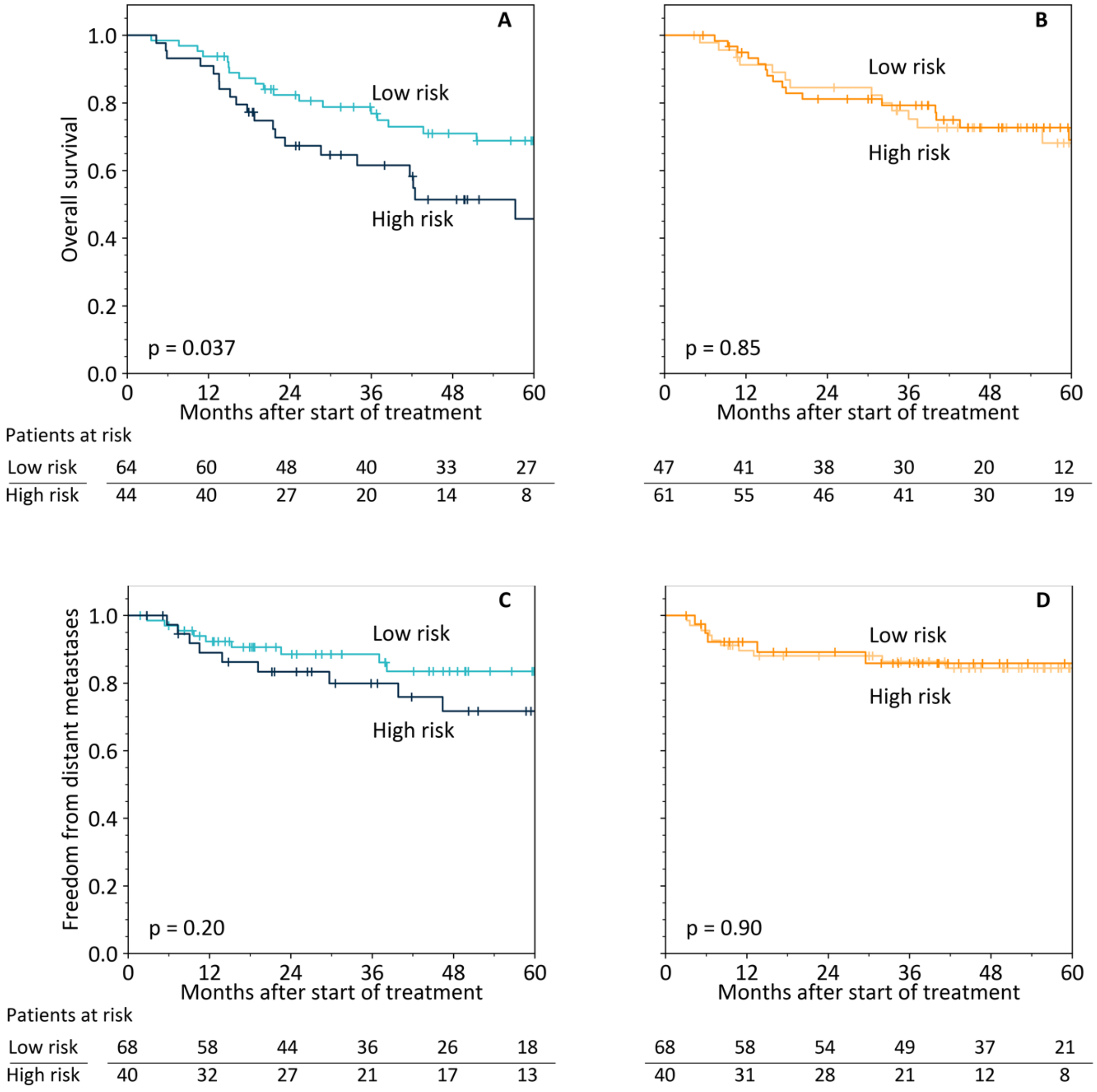

Figure S3. Patient stratification by the 2-metagene signature for overall survival (OS) in the PORT **(A)** and the PORT-C cohort **(B)** and for freedom from distant metastases (DM) in the PORT **(C)** and the PORT-C cohort **(D)**.
